# Prevalence and Manifestations of Dental Ankylosis in Primary Molars Using Panoramic X-rays: A Cross-Sectional Study

**DOI:** 10.3390/children9081188

**Published:** 2022-08-08

**Authors:** Daniela Eşian, Cristina Ioana Bica, Oana Elena Stoica, Timea Dako, Alexandru Vlasa, Eugen Silviu Bud, Denisa Salcudean, Liana Beresescu

**Affiliations:** 1 Faculty of Dental Medicine, University of Medicine and Pharmacy, Science and Technology George Emil Palade of Targu-Mures, 540139 Targu Mures, Romania; 2 Dentoalveolar Surgery Department, Faculty of Dental Medicine, University of Medicine and Pharmacy Iuliu Hatieganu of Cluj-Napoca, 400347 Cluj-Napoca, Romania

**Keywords:** dental ankylosis, infraocclusion, primary molars, dental mobility

## Abstract

Dental ankylosis is a serious condition defined as the process that causes the fusion between the dentin or the cementum of the root and the alveolar bone, with the obliteration of the periodontal ligament becoming progressively replaced by bone tissue. The aim of the study was to determine the prevalence, location, severity, and association of dental ankylosis in primary molars with other dental anomalies such as the agenesis of permanent buds. For this study 150 panoramic x-rays were selected from patients with temporary or mixed dentition, aged six to twelve years old, from a private dentistry office and from the Pediatric Dentistry Department of UMFST in Targu-Mures, Romania. In order to identify the cases with dental ankylosis, the presence and severity of the infraocclusion, displacements of the neighboring teeth, the appearance of the root area, and the relationship with the bone tissue were examined. For evaluation of the categorical data we used Fischer’s exact test and the Chi-squared test and the chosen significance level was set at 0.05. The results showed that the highest percentage of cases with ankylosis was found in the first group (six to nine years old), respectively, with 72% of cases compared with the second group (ten to twelve years old) with 28% of cases. Findings showed that there was no positive association between dental ankylosis and gender, but a strong correlation was found regarding the location on the dental arches. Most cases were identified on the lower arch with a higher percentage in quadrant three. Of the two primary molars, the most affected by ankylosis was the first molar in quadrant three, followed by the second molar, and finally the first molar in quadrant four. Most cases diagnosed with ankylosis had a mild to moderate degree of infraocclusion; therefore, changes in the functional balance of the dental arch and on neighboring teeth were insignificant. There were some differences obtained between our results and studies from the literature, especially regarding the localization in the lower left dental arch, but these differences can be attributed to the number of the subjects selected and from the methodology of dental ankylosis diagnosis. Based on the data obtained, it was concluded that ankylosis is a dental condition which occurs in children in early mixed dentition, especially in the lower arch, with the first primary molar being the most affected tooth. The presence of infraocclusion and the absence of dental mobility, especially during the stage of primary molars’ root resorption, are the main signs which must be followed to make an early diagnosis and prevent further complications.

## 1. Introduction

Dental ankylosis is an eruptive abnormality characterized by the fusion between the dentin or the cementum of the root and the surrounding bone, with the obliteration of the periodontal ligament that will be progressively replaced by bone tissue [1]. It can occur in any stage of tooth eruption, either before the complete eruption in the oral cavity (primary retention) or after the tooth has reached the occlusal plane (secondary retention) [2].

According to Andlaw (1974) the frequency of dental ankylosis has been reported to be between 1.3% and 38.5% [3] and the high incidence of ankylosed primary teeth was seen in children between ages seven and eleven [4,5,6,7].

A simple classification of this dental abnormality can be described as slight, moderate, or severe. According to this classification, “slight” is defined as being between the occlusal surface and the proximal contact; “moderate” being within the occlusal, gingival dimensions of inter-proximal contact points; and “severe” being anywhere below the interproximal contact point [8].

In the last few years, two theories have been proposed in the etiology. The first theory identifies local factors as the main causes and the second theory focuses on genetic factors; however, so far, no responsible genes have been identified [9].

One of the theories states that any disruption in the continuity of the periodontal ligament caused by trauma can lead to the onset of the degenerative processes that will result in the fusion between tooth and bone [10]. If the trauma produced limited lesions, a slight osteoclastic activity will be initiated on the root surface which involves resorption and repair, resulting in healing by depositing new fibers on the cement surface [11].

If the periodontal ligament is completely destroyed, without healing capacity, there will be a gradual replacement of the root with bone and a progressive dental ankylosis will occur; therefore, early diagnosis is essential to prevent complications [12]. Another theory is based on local factors that cause disturbances of the local metabolism that will degenerate the periodontal ligament. The loss of contact between the root surface and the bone results in their fusion.

The diagnosis of dental ankylosis is established following clinical and preclinical investigations and it is essential to be as early as possible [10,13]. The affected tooth is unable to perform the post eruptive movements, thus, it will remain in infraposition with the degree of infraocclusion ranging from 1 mm to complete retention under the gingival tissue, a situation known as severe ankylosis [14,15].

Radiological examination is essential for the diagnosis of ankylosis. Radiological images will show the fusion between the bone and the root surface as well as the absence of periodontal space. Additionally, the roots lose their opacity and, in cases of severe ankylosis, there is no clear delimitation from the surrounding bone [10,12].

Ankylosis of primary molars can cause severe clinical consequences in the growing child including tooth infraocclusion and vertical bone defect, tipping of adjacent teeth into the space of infraocclusion causing loss of arch space, dental asymmetry, midline deviation and impaction of the ankylosed tooth and its successor, supra-eruption of opposing teeth, and deflected path of eruption of successors, with displacement in the form of tipping and ectopic eruption of successors [8,16]. Extensive bony ankylosed primary molars interfere with the exfoliation and eruption of the permanent successors [16,17]. There is currently no therapeutic algorithm of choice but based on the existing studies most cases of dental ankylosis can be treated properly. Therapeutic approach depends on the presence of a permanent bud, diagnosis timing, and severity of infraocclusion [18].

Most authors recommend early extraction of the ankylosed primary tooth and subsequent space management, particularly when patients are in the stages of early mixed dentition [19,20]. The main objective of this study is to identify the prevalence, location, severity, and association of dental ankylosis with agenesis of permanent tooth buds using panoramic x-rays selected from a group of Romanian children.

## 2. Materials and Methods

This study was conducted between October 2019 and May 2021, in accordance with the Declaration of Helsinki and was approved by the Ethics Committee of Human Research (protocol code 019; date of approval was 10 October 2019). The patients were informed about the purpose of the study and their written consent was obtained. The study group consisted of 180 subjects with temporary and mixed dentition. The sample group included boys and girls aged between six and twelve years old selected from a private dentistry office and from the Pediatric Dentistry Department, UMFST in Targu-Mures, Romania. The subjects were involved in the study indirectly by using panoramic x-rays of their cases. The main selection criteria were that subjects had temporary or mixed dentition and at least one quadrant of the support area without destruction, restorations of the crowns, or extractions. The exclusion criteria involved complete destruction of the support areas, poor quality of radiographs such as distortion that did not allow for the identification of different degrees of infraocclusion, which is considered to be the main sign of dental ankylosis.

After evaluation based on the main criteria, only 150 panoramic radiographs were selected: 78 from girls and 72 from boys. The participants’ radiographs were divided into two main age sub-groups as follows:

- Group 1: radiographs from six to nine years old included (*n* = 70);
- Group 2: radiographs from ten to twelve years old included (*n* = 80).

A sample size calculator was used to determine whether the number of subjects included in this study was large enough to generalize the findings regarding the prevalence of dental ankylosis and agenesis of permanent buds. If 50 more patients had been included in the original number of subjects, the sample size would have been optimal.

The diagnosis of dental ankylosis involved radiographic examination. In the study group the diagnosis was informed by the presence of different degrees of infraocclusion and the appearance of adjacent bone (the loss of periodontal space).

The diagnosis of a primary molar with infraocclusion was performed using the occlusal plane and neighboring teeth as references, after which the ankylosed tooth was compared with the corresponding molar in the opposite quadrant. The radiographic aspect of the surrounding bone was not considered because of its variability.

The analysis of the panoramic x-rays was performed twice by two examiners at an interval of at least two weeks, under optimal conditions, using a white-light illuminator and the data obtained were systematized as follows:

- The presence of infraocclusion in primary molars;
- The severity of infraocclusion: slight (less than 1 mm), moderate (over 1 mm), and severe infraocclusion (the occlusal surface is below the level of the contact points);
- The position in the dental arch and in the quadrant;
- The most frequently affected molar;
- The age and sex of the subjects;
- The absence of permanent buds.

## 3. Statistical Analysis

All data was collected in Microsoft Excel work sheets (Microsoft Corporation, 2018, Redmond, WA, USA). The statistical analysis was carried out in GraphPad Prism version 8.0.0 for Windows (GraphPad Software, San Diego, CA, USA). Data normality was determined using the Kolmogorov–Smirnov test. For evaluation of the categorical data, we used Fischer’s exact test and the Chi-squared test. The chosen significance level was set at 0.05.

## 4. Results

A total of 180 panoramic x-rays were initially included in the study and after evaluation based on the inclusion/exclusion selection criteria only 150 radiographs remained, of which 72 were from male patients and 78 were from female patients aged between six and twelve years old, with an average age of 8.2 years. The characteristics of the subjects and the main results are summarized in Table 1.

Out of the 150 radiographs included in the study, only eighteen (*n* = 18) radiographs showed specific signs of dental ankylosis associated with different degrees of infraocclusion. Twelve cases (*n* = 12) of agenesis of the permanent bud (premolars) were found from the total of 150 subjects. No positive association for the prevalence of dental ankylosis was found between genders (*p* = 0.9439) or agenesis of permanent buds (*p* = 0.7673).

**Figure 1:**
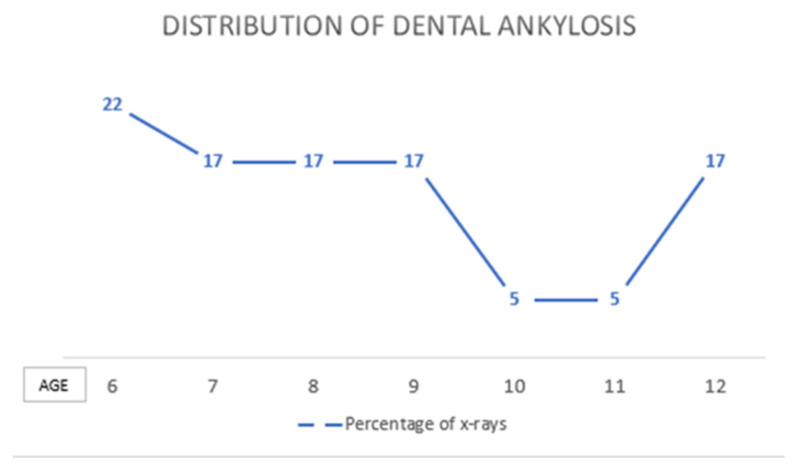
Distribution of dental ankylosis according to the age of the subjects.

With regard to the age group, it was observed that a statistical difference exists between the two groups. The highest percentage of ankylosis was found in the first group (six to nine years old) with thirteen radiographs (*n* = 13) which equates to 72% compared with the second group (aged ten to twelve years old) with five radiographs (*n* = 5) equating to only 28%. The difference between the two age groups was found to be statistically significant (*p* = 0.024). (Table 2, Figure 1).

The highest prevalence of ankylosis occurred in Group 1, in those with early mixed dentition. The rates are as follows: four cases (*n* = 4) at the age of six (22%); followed by the ages of seven, eight, and nine each with three cases (*n* = 3), equating to a percentage of 17% of participants in each of those three ages diagnosed with dental ankylosis.

With regard to the association of ankylosis with other dental anomalies, twelve cases (*n* = 12) of the agenesis of permanent buds were found in the study group. Out of these cases, five (*n* = 5) were boys and seven (*n* = 7) were girls. Eight cases (*n* = 8) with agenesis were simultaneously associated with dental ankylosis, equating to a percentage of 44%. In six subjects (*n* = 6), the agenesis was bilateral equating to 75% of cases, while in the remaining two cases (*n* = 2) the agenesis was unilateral.

A higher percentage of cases was observed in the third quadrant, followed by the fourth quadrant. Therefore, of the total of 30 molars diagnosed with ankylosis: seventeen molars (*n* = 17), or 57% of cases, were located in the third quadrant; twelve molars (*n* = 12), or 40%, were located in the fourth quadrant; a single molar (3%) was located in the second quadrant; and none in quadrant one (Table 3).

Out of the eighteen radiographs (*n* = 18) showing signs of ankylosis, the total number of affected molars was thirty (*n* = 30), of which six subjects (*n* = 6), or 33%, had a single affected molar and twelve subjects (*n* = 12), or 67%, had two affected molars. The distribution on each molar is presented in Table 4.

With regard to the degree of infraocclusion, it was observed that out of the thirty (*n* = 30) molars with ankylosis, seventeen molars (*n* = 17), or 57%, had slight infraocclusion (less than 1 mm); thirteen molars (*n* = 13), or 43%, had moderate infraocclusion (over 1mm); and no molars showed signs of severe infraocclusion (the occlusion plane of the affected molar was located below the contact point).

Due to the fact that most of the cases had a mild infraocclusion, a small percentage of pathological dental migration was observed. Five cases (*n* = 5), or 17%, out of the thirty subjects diagnosed with ankylosis showed this pathological dental migration. Dental ankylosis did not affect the exfoliation process; thus, out of the thirty cases (*n* = 30), only six molars (*n* = 6) show signs of late exfoliation (20%).

## 5. Discussions

This study aimed to evaluate the prevalence, location, severity, and association with agenesis of the permanent bud of dental ankylosis on a group of Romanian subjects with temporary and mixed dentation.

For a precise diagnosis of dental ankylosis, both a clinical and radiological examination is nedeed, because the onset occurs on a single surface of the root and the panoramic radiograph that provides a two-dimensional image is sometimes insufficient for a definite diagnosis [21]. 

In this study, dental ankylosis had a prevalence of 12%, a result very similar to the 10.4% obtained by Zuniga-Tertre et al. in 2004 [22]. However, in another study conducted in 2018 by Venza N., a prevalence of 2.8% was obtained [23]. These differences are likely due to the major influence that the selection criteria and the ethnicity of the studied populations had on the results.

According to the results, dental ankylosis occurred with a higher prevalence in the early stage of mixed dentition in children aged 6 to 9 years. This result is similar to existing studies [24,25]. The prevalence of this condition is ten times higher in decidual dentition than in permanent dentition, with a percentage of 1.3–8.9% in children aged 6–10 years in the first stage of mixed dentition, according to the literature [10]. Zuniga-Tertre et al. (2004) observed a maximum prevalence between six to eight years and another study conducted in 2009 by Loriato found the maximum incidence occurred between seven to nine years [26]. Since this study a criterion for diagnosis of dental ankylosis was the presence of an already infraoccluded molar, the exact moment of onset cannot be determined.

Regarding the distribution by gender, the findings showed that there were no statistically significant differences between genders for the prevalence of ankylosis, which is similar to most existing studies where there was no predilection for boys or girls. However, there are some studies with different results. For example, a study conducted in 2017 reports a higher incidence in girls where out of 402 subjects with ankylosis 238 were girls and 164 boys [27].

The present results showed that lower molars were much more affected by ankylosis than upper molars, which is in accordance with the literature as it supports the finding that this condition primarily affects the lower dental arch. A very representative example is a study conducted in 2018 by Venza N. et al. on 4706 subjects, which identified 189 cases in the mandible and only 36 in the upper jaw [23]. According to other studies, the lower primary molars are ten times more affected compared to the upper molars.

Some contradiction exists between the results of this study and other existing findings. Specifically with regard to the quadrant most frequently affected by ankylosis. In this study a higher prevalence of ankylosis was found in quadrant three, followed by quadrant four. However, most studies support the conclusion that this condition does not affect a particular quadrant and that the frequency was relatively equal between the right and the left side [23,28]. There are also a small number of studies showing that the incidence of ankylosis is higher in the primary right molars [22,27]. However, it is possible that these differences may be influenced by the number of subjects included in the study. If 50 more patients would have been included in the original number of subjects, the results would have been more accurate.

The present results show that the first lower primary molar was the most frequently affected with higher incidence in quadrant three. This was followed by the second lower primary molar in quadrant three and then the first and second upper molars.

Another aspect that was noticed in this study is the age of those affected. Ankylosis of the primary first molar occurred at a younger age compared to the second molar.

This result was different from most studies as the second primary molar has been reported to be more often affected [23,26]. However, an earlier study by Zuniga-Tertre et al. supports our results. This variability can probably be attributed to the number of subjects included in the study, but also to the fact that the diagnosis of dental ankylosis was based only on infraoccluded primary molars. The degree of infraocclusion was, in most cases, slight or moderate and, thus, it is possible to have missed a diagnosis of ankylosis in another molar. Moreover, in the present study, the diagnosis of dental ankylosis was based only on radiological criteria; therefore, subjects undergoing orthodontic treatment were not identified which could have influenced the variability of the results.

According to the results, a higher percentage of cases with ankylosis had mild to moderate infraocclusion and there were no cases with severe infraocclusion. These findings correspond to most existing studies. For example, in their study Zuniga-Tertre et al. reported a slight infraocclusion in 69.7% of cases with ankylosis [22].

Ankylosis of primary molars almost always causes infraocclusion compared to the adjacent teeth because the ankylosed tooth becomes immobile, which is related to the eruption changes that occur with growth [29]. The degree of infraocculsion depends mainly on the stage of occlusal development at the time when ankylosis occurs, causing negative effects such as loss of space or the onset of carious lesions due to difficult oral hygiene conditions [30,31]. Considering these factors, it is recommended that all cases with infraoccluded primary molars be monitored carefully to ensure normal exfoliation, especially when the permanent tooth bud is present.

In this study, agenesis of a premolar bud associated with ankylosis occurred in eight out of eighteen radiographs. Six cases (75%) were bilateral, and the two remaining cases were unilateral. This implies that there is an indirect correlation between the two dental anomalies with different etiological factors. A study published in 2018 [28] reports that 65.7% of subjects with premolar agenesis also had ankylosis of primary molars and considered that hypodontia represents a possible etiological factor in ankylosis of these molars. In another study [32], it was observed that the association of premolar agenesis and ankylosis of the second primary molars had a prevalence of 18%, which is contrary to the hypothesis that in the etiology of ankylosis the main role is played by local factors and supports the genetic origin of this condition.

### Limitations of the Study

We must mention that the study had some limitations regarding the size of the sample group and the methodology of dental ankylosis diagnosis, which involved only radiological features and no clinical examination. This study is ongoing and further results will be published.

## 6. Conclusions

In the present study, the prevalence of dental ankylosis was higher in the early stage of mixed dentition (six to nine years old) and a higher percentage of cases was observed in the third quadrant, especially in the first primary molars. More than half of the subjects diagnosed with ankylosis were girls, but the difference in distribution by gender was not statistically significant and in most cases the degree of infraocclusion was slight to moderate.

The variations of the results depend on the populations selected for the study, the methodology of the study, and the inclusion/exclusion criteria.

This study offers a good foundation for future research on this topic and cannot be ignored, as other authors have had similar findings using larger sample groups.

## Figures and Tables

**Table 1:** The characteristics of the subjects analyzed in the study.

Characteristics	Frequency	Percentage
	(*n*)	(%)
Radiographs	150	
**Gender**		
Boys	72	48
Girls	78	52
**Age group**		
6 to 9 years	70	46.6
10 to12 years	80	53.3
**Ankylosis**	18	
Boys	8	44
Girls	10	56
**Agenesis of** **permanent bud**	12	
Boys	5	41.66
Girls	7	58.33

**Table 2:** Distribution of dental ankylosis according to age.

Group	1 (Six to Nine)				2 (Ten to Twelve)			Total
Age (years)	6	7	8	9	10	11	12	
Number of radiographs (*n*)	4	3	3	3	1	1	3	18
Percentage (%)	22	17	17	17	5	5	17	100

**Table 3:** Distribution of ankylosis based on location.

Location	2nd Quadrant	3rd Quadrant	4th Quadrant	Upper Jaw	Lower Jaw
Number of molars (*n*)	1	17	12	1	29
Percentage (%)	3	57	40	3	97

**Table 4:** Number of ankylosis cases on each primary molar.

Primary Molar	64	74	75	84	85
Number of cases	1	11	7	6	5
Percentage	3	37	23	20	17

## Data Availability

All data regarding this manuscript can be checked with the corresponding author at alexandru.vlasa@umfst.ro.

## References

[1] Alruwaithi M., Jumah A., Alsadoon S., Berri Z., Alsais M. (2017). Tooth ankylosis and its orthodontic implication. J. Dent. Med. Sci..

[2] Yoon G., Lee N., Lee S., Jih M. (2020). Eruption guidance of horizontally impacted permanent first molar with primary retention of primary second molars: Case reports. J. Korean Acad. Pediatric Dent..

[3] Messer L.B., Cline J.T. (1980). Ankylosed primary molars: Results and treatment recommendations from an eight-year longitudinal study. Pediatric Dent..

[4] Kurol J. (1981). Infraocclusion of primary molars: An epidemiologic and familial study. Community Dent. Oral Epidemiol..

[5] Noble J., Karaiskos N., Wiltshire W.A. (2007). Diagnosis and management of the infraerupted primary molar. Br. Dent. J..

[6] Rune B., Sarnos K.V. (1984). Root resorption and submergence in retained deciduous second molars. Eur. J. Orthod..

[7] Steigman S., Koyoumdjisky-Kaye E., Matrai Y. (1973). Submerged deciduous molars in the preschool children: An epidemiological survey. J. Dent. Res..

[8] Rubenstein L.K., Lindauer S.J., Issacson R.J. (1991). Development of supernumerary premolars in an orthodontic population. Oral Surg. Oral Med. Oral Pathol..

[9] Hua E., Thomas M., Bhatia S., Bowkett A., Merrett S. (2019). To extract or not to extract? Management of infraoccluded second primary molars without successors. Br. Dent. J..

[10] Cardozo M.A., Hernández J.A. (2015). Diagnóstico y manejo de la anquilosis dentoalveolar. Rev. Odontopediatría Lat..

[11] Lauridsen E., Andreasen J.O., Bouaziz O., Andersson L. (2020). Risk of ankylosis of 400 avulsed and replanted human teeth in relation to length of dry storage: A re-evaluation of a long-term clinical study. Dent. Traumatol..

[12] Thumbigere-Math V., Alqadi A., Chalmers N.I., Chavez M., Chu E.Y., Collins M.T., Ferreira C.R., FitzGerald K., Gafni R.I., Gahl W.A. (2018). Hypercementosis associated with ENPP1 mutations and GACI. J. Dent. Res..

[13] Lopes Rosa D.C., Simukawa E.S., Alvares Capelozza A.L., Perri de Carvalho P.S., Vicente Rodrigues M.T. (2019). Alveolodental ankylosis: Biological bases and diagnostic criteria. RGO Rev. Gaúcha Odontol..

[14] Barletta M.C., Omedeiros V., Fsilveira A.C., Fernandes N., Suzana Cruz M.L.M.F. (2018). Severe dentoalveolar ankylosis of primary molars: Therapeutic conducts. Open J. Clin. Med. Case Rep..

[15] Becker A., Karnel-R’em R.M. (1992). The effects of infraocclusion: Part 1. Tilting of the adjacent teeth and local space loss. Am. J. Orthod. Dentofac. Orthop..

[16] Biederman W. (1968). The problem of the ankylosed tooth. Dent. Clin. N. Am..

[17] Konstat M.M., White G.E. (1975). Ankylosed teeth: A review of the literature. J. Mass Dent. Soc..

[18] Jenkins F.R., Nichol R.E. (2008). Atypical retention of infraoccluded primary molars with permanent successor teeth. Eur. Arch. Paediatr. Dent..

[19] Cozza P., Gatto R., Mucedero M. (2004). Case report: Severe infraocclusion ankylosis occurring in siblings. Eur. J. Paediatr. Dent..

[20] Belanger G.K., Strange M., Sexton J.R. (1986). Early ankylosis of a primary molar with self-correction: Case report. Am. Acad. Pediatr. Dent..

[21] Ducommun F., Bornstein M.M., Bosshardt D., Katsaros C., Dula K. (2018). Diagnosis of tooth ankylosis using panoramic views, cone beam computed tomography, and histological data: A retrospective observational case series study. Eur. J. Orthod..

[22] Del Zúñiga-Tertre M.P., Lucavechi-Alcayaga T., Barbería Leache E. (2004). Distribución y gravedad de las infraoclusiones de molares temporales. Rev. Cons. Odontológos Estomatólogos.

[23] Venza N., Danesi C., Cretella Lombardo E., Gazzani F. (2018). Infraocclusion of deciduous molars: A retrospective analysis of prevalence, characteristics and association with other dental anomalies. Oral Implantol..

[24] Silvestrini Biavati A., Signori A., Castaldo A., Matarese M., Migliorati G. (2011). Incidence and distribution of deciduous molar ankylosis, a longitudinal study. Eur. J. Paediatric Dent..

[25] Barbosa Loriato L., Wilson Machado A., Quiroga Souki B., Junqueira Pereira F. (2009). Late diagnosis of dentoalveolar ankylosis: Impact on effectiveness and efficiency of orthodontic treatment. Am. J. Orthod. Dentofac. Orthop..

[26] Álvaro G.S., Jorge C.D., Esther O.F., Víctor S.S. (2017). Study of dental ankylosis in a child population. Rev. Complut. De Cienc. Vet..

[27] Odeh R., Mihailidis S., Townsend G., Lähdesmäki R., Hughes T., Brook A. (2016). Prevalence of infraocclusion of primary molars determined using a new 2D image analysis methodology. Aust. Dent. J..

[28] Aristidis Arhakis E.B. (2016). Etiology, diagnosis, consequences and treatment of infraoccluded primary molars. Open Dent. J..

[29] American Academy of Pediatric Dentistry (2020). Management of the developing dentition and occlusion in pediatric dentistry—Guidelines. Am. Acad. Pediatric Dent..

[30] Krakowiak F.J. (1978). Ankylosed primary molars. J. Dent. Child..

[31] Na-Young O., Soon-Hyeun N., Jae-Sik L., Hyun-Jung K. (2020). Delayed spontaneous eruption of severely infraoccluded primary second molar: Two case reports. J. Clin. Pediatric Dent..

[32] Choi S.J., Woo Lee J., Hyun Song J. (2017). Dental anomaly patterns associated with tooth agenesis. Acta Odontol. Scand..

